# Beyond the Rash: The Devastating Course of Meningococcal Purpura Fulminans in an Unvaccinated Young Adult

**DOI:** 10.7759/cureus.87617

**Published:** 2025-07-09

**Authors:** Hannah R Malinosky, Kristen Santiago

**Affiliations:** 1 Internal Medicine, Texas Health Resources, Dallas, USA

**Keywords:** acute infectious purpura fulminans, alcohol use disorder (aud), covid and purpura fulminans, medical dermatology, meningococcemia, neisseria meningitidis, vaccine preventable diseases

## Abstract

Purpura fulminans (PF) is a life-threatening thrombotic disorder characterized by rapidly progressive cutaneous hemorrhagic necrosis, disseminated intravascular coagulation (DIC), and multi-organ failure. Most commonly associated with meningococcemia, PF requires early recognition for optimal outcomes. This report presents the case of a 26-year-old immunocompetent but unvaccinated male who developed PF secondary to meningococcemia, complicated by refractory septic shock, multi-organ failure, and extensive tissue necrosis. His clinical course underscores the aggressive nature of this condition, the critical need for early recognition, and the importance of primary prevention through meningococcal vaccination. The patient’s unvaccinated status highlights persistent gaps in vaccination uptake, particularly among individuals outside of university settings. Co-infection with COVID-19 and underlying alcohol use disorder may have contributed to immune dysregulation and increased disease severity. This case also raises awareness of the potential role of COVID-19 in the pathogenesis of meningococcemia. PF remains a dermatologic and hematologic emergency with high mortality, necessitating rapid diagnosis and a multidisciplinary treatment approach. Strengthening vaccination education and advocacy is essential to reduce the burden of PF and its complications.

## Introduction

Purpura fulminans (PF) is a rare but critical dermatologic emergency that requires prompt diagnosis and management, accounting for less than 0.5% of septic shock cases [1]. It is a subtype of disseminated intravascular coagulation (DIC) characterized by rapidly progressive cutaneous hemorrhagic necrosis and frequently leads to circulatory collapse [2, 3]. The hallmark of clinical presentation includes widespread purpuric lesions, petechiae, and necrosis, often associated with systemic signs of sepsis, multiorgan dysfunction, and coagulopathy [3]. 

PF exhibits a bimodal distribution, primarily affecting children aged 1-3 years and adolescents aged 16-18 years [4]. The latter peak is often associated with *Neisseria meningitidis *(*N. meningitidis*) infection, as young adults serve as reservoirs for transmission via respiratory secretions. The bacterium disproportionately affects individuals in communal living environments, such as university dormitories, military barracks, detention centers, homeless shelters, and other group home environments [5]. In the United States, while nearly all college students receive the quadrivalent meningococcal conjugate vaccine (MenACWY), only 38-75% of individuals not attending university are vaccinated, with rates varying by factors such as geographical location [5]. 

PF stems from dysregulated inflammation and endothelial injury, leading to microvascular thrombosis and tissue ischemia [2]. Early recognition of its dermatologic manifestations is critical, as delayed management increases morbidity and mortality [6]. Treatment requires a multidisciplinary approach involving infectious disease (ID) specialists, critical care teams, and surgical intervention for tissue debridement or amputation in severe cases [2]. Despite these aggressive interventions, PF incurs a 50% mortality rate, and survivors often experience significant morbidity, including scarring and limb loss [4, 7]. 

This report presents a case of a 26-year-old immunocompetent but unvaccinated male who developed PF secondary to meningococcemia, complicated by refractory septic shock, multiorgan failure, and extensive tissue necrosis. His clinical course emphasizes the aggressive nature of this condition, the importance of early recognition, and the need for improved primary prevention through meningococcal vaccination education. 

## Case presentation

A 26-year-old unvaccinated male with a history of alcohol use disorder was transferred from a local emergency department for a higher level of care after presenting with fever and hypotension. His symptoms began one week prior with fever, chills, and myalgias, progressing to neck stiffness, nausea, and vomiting, and a rapidly developing rash. He reported recent exposure to COVID-19 and unprotected sexual intercourse.

**Figure 1:**
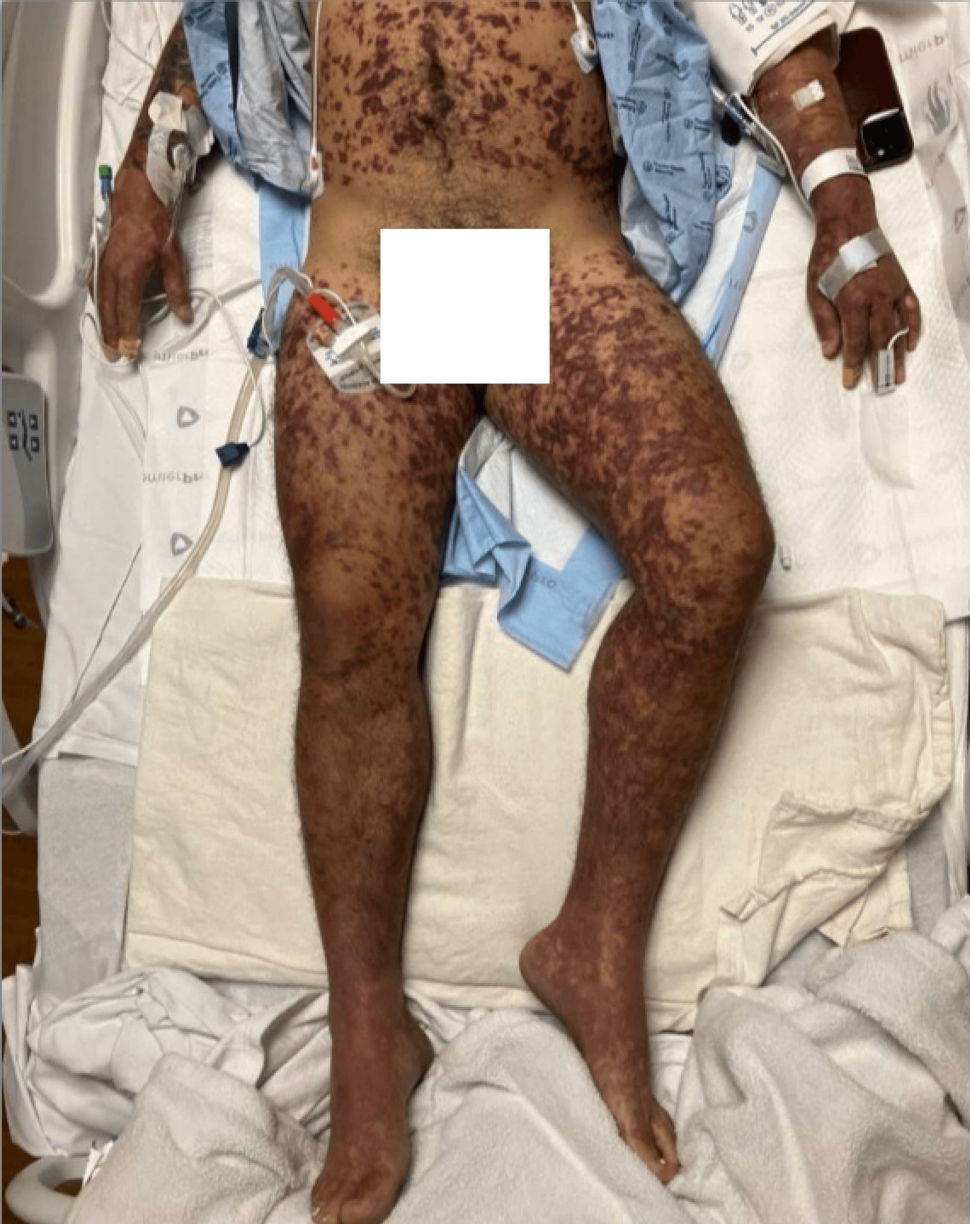
Diffuse, purpuric rash with violaceous macules and patches in a reticular pattern extending from the trunk outwards to the bilateral upper and lower extremities (hospital Day 1).

On arrival, he was febrile (38°C), hypotensive (78/53 mmHg), and tachycardic (185 bpm). Physical examination revealed a violaceous, reticular rash with purpura and petechiae involving the bilateral upper and lower extremities, abdomen, and chest, consistent with evolving PF (Figure 1). Laboratory findings included acute kidney injury, pancytopenia, and coagulopathy. Complement levels were low (Table 1). Abdominal computed tomography (CT) scan showed no splenic abnormalities, and lumbar puncture revealed an elevated white blood cell count (21/µL). 

**Table 1:** Laboratory results on admission with reference values. FEU: fibrinogen equivalent units

Laboratory test	Value	Reference Value
Creatinine	1.6 mg/dL	0.72-1.25 mg/dL
White blood cell count	2.06 k/µL	3.98-9.57 k/uL
Hemoglobin	10.6 g/dL	13.0-17.0 g/dL
Platelet count	55 k/µL	130-140 k/uL
Prothrombin time	25.9 seconds	11.9-15.0 seconds
Partial thromboplastin time	52.5 seconds	23.0-35.4 seconds
International normalised ratio	2.3	0.9-1.2
D-dimer	>20 ug/mL FEU	<=0.50 ug/mL FEU
Fibrinogen	159 mg/dL	204-488 mg/dL
Complement component 3	49 mg/dL	82.0-185.0 mg/dL
Complement component 4	9.2 mg/dL	15.0-53.0 mg/dL

The patient was admitted to the intensive care unit with septic shock and started on broad-spectrum antibiotics, including vancomycin and ceftriaxone, later broadened to piperacillin-tazobactam, alongside stress-dose steroids and aggressive resuscitation measures. At that time, he also tested positive for COVID-19 and was initiated on remdesivir. 

Despite aggressive treatment, his condition deteriorated. His hypotension worsened, requiring vasopressor support. On hospital Day 2, he suffered a cardiac arrest with pulseless electrical activity, progressing into ventricular tachycardia, necessitating defibrillation and various other advanced cardiac life support interventions. Return of spontaneous circulation was achieved, but he remained critically ill and ventilator-dependent. 

The rash progressed rapidly, with expanding areas of necrosis, hemorrhagic bullae, and ulceration, necessitating wound care consultation. He required multiple transfusions of platelets, fresh frozen plasma, and cryoprecipitate for persistent DIC. Blood cultures grew beta-lactamase-producing *N. meningitidis*, prompting antibiotic adjustment to meropenem per the ID service.

**Figure 2:**
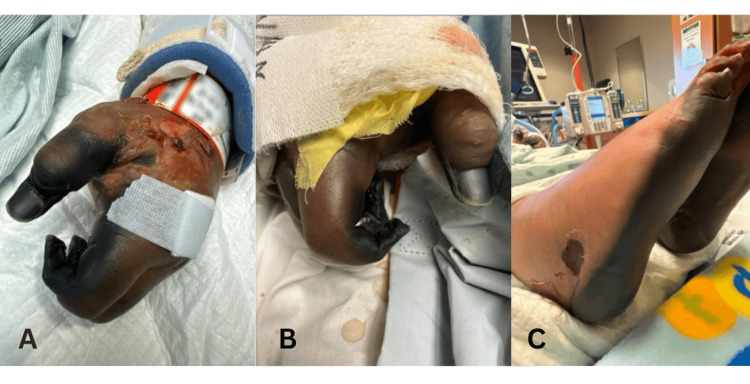
(A) Left hand with severe necrosis of the digits and ulcerations and bullae on the dorsal surface with superficial crusting (hospital Day 18); (B) right hand with severe necrosis of digits (hospital Day 19); (C) bilateral feet with necrosis of dorsal surfaces and ulceration to right lateral foot (hospital Day 19).

The patient developed renal failure requiring continuous renal replacement therapy, acute liver failure, and refractory hypotension. His distal extremities became necrotic (Figure 2), ultimately necessitating consultation from vascular surgery, who recommended bilateral below-the-knee and finger amputations. The patient's prolonged critical illness, recurrent fevers, and agitation hindered extubation attempts. Given his poor prognosis, his family opted for comfort measures. He was extubated on hospital day 26 and passed away the following day. The patient's close contacts, including family members and roommates, received chemoprophylaxis on admission, and those who were unvaccinated received appropriate vaccination. 

## Discussion

PF is a dermatologic and hematologic emergency characterized by intravascular thrombosis leading to hemorrhagic infarction of the dermis [8]. This case highlights the classic PF rash, which progresses from erythematous, punctate macules and petechiae to widespread purpura and hemorrhagic necrosis, sometimes overlying a livedoid pattern or accompanied by hemorrhagic vesicles and bullae, often within 24 hours [4]. When presented with a purpuric, petechial rash, differential diagnoses include autoimmune vasculitides, thrombotic microangiopathies, immune thrombocytopenic purpura, calciphylaxis, cryoglobulinemia, Stevens-Johnson syndrome/toxic epidermal necrolysis, and warfarin-induced skin necrosis. 

The etiology of PF is multifactorial, with bacterial endotoxins playing a significant role in aberrant activation of the coagulation cascade [4, 6, 8]. *N. meningitidis* is particularly potent in inducing this response and is more commonly seen in immunocompromised or asplenic patients [4]. Although this patient was immunocompetent, his concurrent COVID-19 infection and alcohol use disorder likely predisposed him to more severe illness. Chronic alcohol abuse has been reported to be associated with increased illness severity and higher rates of complications [9], while COVID-19 infection increases the risk of thromboembolic events and exacerbates coagulopathies [10].

Social history, including education level, living environment, and vaccination status, is essential in assessing risk [5]. The MenACWY vaccine consists of a primary dose at age 11-12 and a booster dose at age 16. While this vaccine is widely administered among college students, uptake remains suboptimal in non-university populations [5]. The newer MenB (Meningococcal group B) vaccine, which covers the predominant serogroup responsible for invasive meningococcal disease in young adults, remains underutilized [5]. These gaps likely result from insufficient education and encouragement regarding vaccination. 

Unfortunately, this patient presented a week after symptom onset, by which time he had already developed extensive purpura. PF carries a mortality rate greater than 50%, and survivors often face devastating sequelae [11]. However, early recognition and management of PF can reduce morbidity, as early surgical debridement has been shown to improve outcomes [2]. This case underscores the need for heightened awareness, early intervention, and aggressive management of PF. Vaccination education and advocacy are critical in reducing disease burden. 

## Conclusions

PF is a devastating disease. Early identification, prompt intervention, and increased vaccination coverage are essential to improving outcomes and reducing the incidence of this life-threatening condition. Given rising vaccine hesitancy and potential funding challenges in the United States, recognizing PF and identifying individuals at higher risk is increasingly important to prevent fatal outcomes.
